# Cluster channel equalization using adaptive sensing and reinforcement learning for UAV communication

**DOI:** 10.7717/peerj-cs.2557

**Published:** 2024-12-13

**Authors:** Xin Liu, Shanghong Zhao, Yanxia Liang, Shahid Karim

**Affiliations:** 1 School of Information and Navigation, Air Force Engineering University, Xi’an, China; 2 School of Information Engineering, Xi’an Eurasia University, Xi’an, China; 3 School of Communications & Information Engineering, Xi’an University of Posts & Telecommunications, Xi’an, China

**Keywords:** Blind equalization, U-Net, Fuzzy reinforcement *Q* learning, Adaptive perception, Dynamic equalization

## Abstract

Aiming to address the need for dynamic sensing and channel equalization in UAV cluster communication environments, this article introduces an equalization algorithm based on a U-Net model and fuzzy reinforcement *Q*-learning (U-FRQL-EA). This algorithm is designed to enhance the channel sensing and equalization capabilities of UAV communication systems. Initially, we develop a U-Net-based signal processing algorithm that effectively reduces acoustic noise in UAV communication channels and enables real-time, accurate perception of channel states by automatically learning channel features. Subsequently, we enhance fuzzy reinforcement *Q*-learning by incorporating a fuzzy neural network to approximate the *Q*-values and integrating this approach with the allocation strategy of wireless sensing nodes. This enhancement not only improves the accuracy of *Q*-value approximation but also increases the algorithm’s adaptability and decision-making ability in complex channel environments. Finally, we construct the U-FRQL-EA equalization algorithm by combining the improved U-Net model with fuzzy reinforcement Q-learning. This algorithm leverages the U-Net model to sense channel states in real time and intelligently adjusts data forwarding strategies based on fuzzy values generated by the fuzzy reinforcement *Q*-learning. Simulation results demonstrate that the U-FRQL-EA algorithm effectively reduces the system’s bit error rate, enhances communication quality, and optimizes network resource utilization, offering a novel solution for improving the performance of uncrewed aerial vehicle communication systems.

## Introduction

Since its inception in 1914, the unscrewed aerial vehicle (UAV) has evolved significantly in both military and civilian applications. UAVs, as autopilot vehicles, are now extensively employed in military reconnaissance, disaster rescue, flood control, drought relief, border inspection, and other complex tasks due to their autonomous navigation capabilities and the ability to operate without direct human intervention. Technological advancements have enabled UAVs to become increasingly miniaturized, highly mobile, and well-concealed, enhancing their value for operations in complex environments (Chukwu & Sweetman, 2024).

In the realm of UAV communication, the cooperative operation of UAV clusters has emerged as a significant trend. However, UAV cluster communication channels exhibit multipath and time-varying characteristics. Traditional adaptive equalization techniques require periodic transmission of training sequences to track channel variations, which not only diminishes communication efficiency but also escalates system complexity and resource consumption (Iqbal et al., 2024). Conversely, adaptive sensing technologies offer precise channel parameters for equalization by monitoring and predicting channel states in real time. In UAV cluster communication, adaptive sensing can provide real-time monitoring of inter-UAV channel states and dynamically adjust equalization strategies according to channel variations, thereby addressing the multipath and time-varying nature of the channel more effectively (Banafaa et al., 2024). Reinforcement learning  (Shuford, 2024), an artificial intelligence technique, enables computers to discover optimal behaviors in unknown environments through continuous trial, error, and feedback optimization. Applied to UAV cluster channel equalization, reinforcement learning can enhance parameter optimization and strategy selection for equalizers, facilitating adaptive adjustment and improving communication quality by learning and adapting to channel changes. Consequently, leveraging adaptive perception and reinforcement learning can significantly enhance the reliability and efficiency of UAV cluster communication (Alam & Moh, 2024).

However, in UAV trunked communication, equalization processing in complex multipath channels presents a significant research challenge. Effective equalization processing in high-speed environments can track and adapt to real-time channel changes, suppressing multipath interference and inter-code interference, thereby enhancing the reliability and stability of UAV trunked communication. The multipath and time-varying characteristics of UAV communication channels can degrade signal quality, leading to low channel measurement accuracy. Additionally, cluster channel equalization in UAV communication is crucial for ensuring efficient and reliable interactions between UAVs and between UAVs and ground equipment. Given the high mobility and flexibility of UAVs, their communication channels are influenced by factors such as UAV position, speed, direction, and physical environment. Variations in these factors can cause fluctuations in channel quality, impacting communication reliability and efficiency. In the complex communication environment, UAV cluster is vulnerable to many kinds of signal interference from outside and inside, such as electromagnetic interference, same frequency interference and malicious interference. These interferences not only affect the stability and reliability of communication links, but also may lead to data transmission errors, control instruction failure and other serious consequences. Therefore, it is the key to ensure the safe and stable operation of UAV cluster communication system to study how to monitor the channel state in real time through adaptive sensing technology and dynamically adjust the channel equalization strategy combined with reinforcement learning algorithm to effectively deal with and reduce the impact of signal interference. At the same time, it is necessary to dynamically adjust the communication parameters according to the channel state when sensing the channel state. This study integrates adaptive sensing, reinforcement learning, and blind equalization techniques to offer a novel solution for UAV cluster communication and to advance the application and development of UAV technology across various fields. The specific contributions of this paper are as follows:

(1) Development of the U-Net-Based Signal Processing Algorithm: A signal processing algorithm based on the convolutional neural network (CNN) U-Net is constructed to accurately predict channel changes by automatically learning channel features. This approach enables acoustic noise reduction and real-time sensing of the UAV communication channel state.

(2) Enhanced fuzzy reinforcement *Q*-learning: The fuzzy reinforcement *Q*-learning method is improved by using a fuzzy neural network to approximate the *Q*-value. This improvement integrates the *Q*-value approximation with the wireless sensing node allocation process, achieving high-precision *Q*-value approximation.

(3) Construction of the U-FRQL-EA Equalization Algorithm: The U-FRQL-EA algorithm combines the enhanced U-Net model with fuzzy reinforcement *Q*-learning. The U-Net model facilitates real-time channel state sensing, while data forwarding is managed according to fuzzy values derived from fuzzy reinforcement *Q*-learning. If data transmission fails, the node automatically reduces its fuzzy value to zero, minimizing network blockage and enabling dynamic network equalization.

## Related Works

As a pivotal technology in the field of information, wireless communication technology is highly valued by researchers and scholars. However, the multipath propagation of wireless signals can cause inter-symbol interference and inter-code crosstalk, adversely affecting transmission efficiency and communication quality. Unlike traditional adaptive equalization techniques that require extensive training sequences, blind equalization (Liu, Wang & Wei, 2024) adjusts system parameters adaptively without relying on training sequences, thereby mitigating inter-code crosstalk and enhancing communication quality. As blind equalization techniques have evolved, researchers have increasingly focused on applying these techniques to various scenarios and modulation methods. Currently, blind equalization techniques can be categorized into four main types based on their algorithmic principles: Bussgang-type blind equalization algorithms (Wang et al., 2024), blind equalization algorithms based on signal detection (Chen, Dong & Xiao, 2024), blind equalization algorithms based on higher-order spectra (Yang et al., 2024), and blind equalization algorithms based on neural networks (Shwetha, Priyatham & Gangadhar, 2024).

The benefits of incorporating deep learning into communication systems are becoming increasingly evident as the applications of AI and deep learning expand. In the realm of equalization techniques, neural networks are anticipated to overcome the limitations of traditional blind equalizers due to their proficiency in solving nonlinear problems. Moreover, the highly cascaded nature of neural networks contributes to faster learning and superior automatic training, resulting in enhanced equalization performance, including improved noise resistance and accuracy, compared to traditional equalizers.

The earliest neural network model applied to blind equalization algorithms was the multilayer perceptron (MLP) (Naseer & Jalal, 2024). This model consists of an input layer, an output layer, and one or more hidden layers, facilitating nonlinear mapping from input to output signals and thereby achieving nonlinear equalization. The Back Propagation (BP) algorithm (Hou, Zhang & Du, 2024) is typically employed for training MLPs, primarily due to its widespread adoption and ease of hardware implementation. However, MLP equalizers are characterized by long and uncertain training times, as well as challenges in structural selection.

Literature (Chew, He & Zhang, 2024) integrates radial basis function (RBF) neural networks (Jitchaijaroen et al., 2024) with transversal equalizers, employing the recursive least squares (RLS) algorithm (Lai & Bernstein, 2024) to update weights. This approach reduces the number of basis functions needed compared to traditional methods and demonstrates superior performance in handling nonlinear channels. Literature (Chen et al., 2024a) introduces an RBF neural network equalization algorithm optimized using an immune algorithm. The immune algorithm, a computational technique for solving combinatorial search and optimization problems, is used to optimize the parameters of the implicit layer in the neural network structure. This optimization process identifies appropriate weights, resulting in improved equalization performance. Literature (Zhao et al., 2024) presents a CNN-based equalization algorithm, CNN-FA. This algorithm incorporates a CNN structure consisting of an input layer, two convolutional layers, a fully connected layer, and a softmax layer between the output layers to achieve effective equalization. Literature (Shyni & Chitra, 2024) proposes a complex convolutional neural network equalization algorithm with a hybrid connection structure. This structure includes input layers, multiple convolutional units, and output layers. Each convolutional unit consists of a convolutional layer, a batch normalization layer, and a Leaky-ReLU activation function. This approach allows the CNN to operate in the complex-valued domain and has been experimentally shown to outperform traditional equalization algorithms.

In summary, with the advancement of artificial intelligence and machine learning, neural network-based equalization technology has become a significant research area. Depending on the application environment, different neural network structures can be selected for equalization tasks. Additionally, as research into this technology progresses, further optimization of these equalizers can be achieved, leading to the development of blind equalizers with enhanced performance.

## Methods

### Improved signal noise reduction algorithm for U-net model

**Figure 1:**
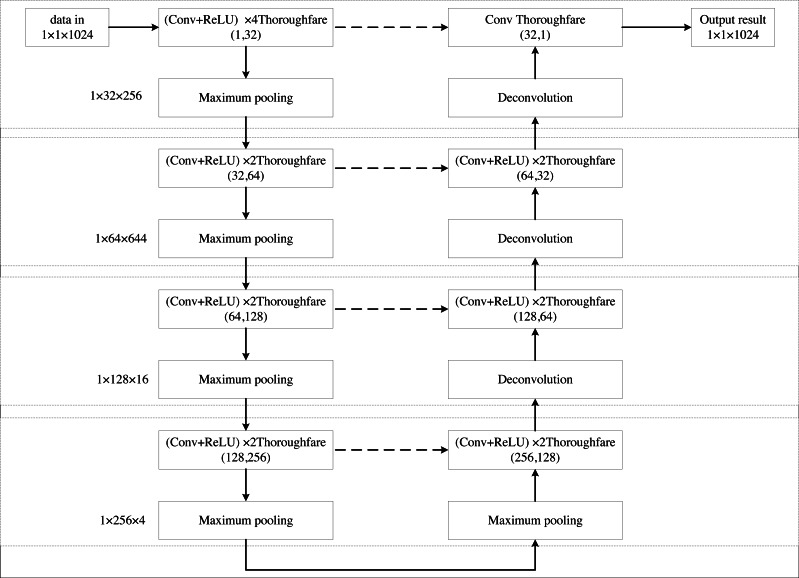
The network structure diagram of the improved U-Net model.

The U-Net network (Chen et al., 2024b) is a fully convolutional network designed around encoding and decoding operations. It processes input images that have undergone a mirroring operation, with each image sized at 572 × 572 pixels. Given that the input layer of the U-Net network is typically fed with image data, and the UAV acoustic magnetoacoustic signals discussed in this paper are one-dimensional, modifications to the U-Net architecture are necessary. The structure of the improved U-Net model is illustrated in Fig. 1.

 In the U-Net model, the original input size is 572 × 572 pixels, designed for 2D image data. Since the acoustic signal being processed is one-dimensional, the model needs adaptation. Specifically, the 2D image input is transformed into a 1 × 1 × 1,024 one-dimensional signal input. The one-dimensional acoustic signals are inherently linear, hence the utilization of one-dimensional convolution kernels (such as 1 × 5) enables direct convolution operations on these signals, eliminating the need for meaningless expansion in another dimension as seen in two-dimensional convolutions. This design facilitates a more efficient capture of local features within the signal. By setting the width of the 1 × 5 convolution kernel, we can grasp the dependency relationships spanning a broader range within the signal without introducing excessive computational complexity. This is particularly crucial for one-dimensional acoustic signals, as some of their features may extend across multiple sampling points.

In the decoder section of the U-Net, the deconvolution operation serves to upsample the signals, restoring their dimensionality. Employing a 1 × 4 deconvolution kernel achieves a two-fold upsampling effect. Furthermore, by adjusting the size of the deconvolution kernel, we can control the width of the feature maps post-upsampling, ensuring seamless alignment with corresponding feature maps from the Encoder section during concatenation. This is pivotal for maintaining the structural symmetry and performance of the model.By implementing these modifications to the U-Net architecture, we enable it to process one-dimensional acoustic signals effectively.

The U-net model is renowned for its distinctive U-shaped architecture, which comprises two primary components: an encoder and a decoder, both symmetrically mirrored in terms of layer depth. The encoder section progressively downsamples the input through multiple layers of convolution and pooling operations, extracting high-level features while reducing spatial resolution. Conversely, the decoder section reverses this process by employing multiple layers of deconvolution (transposed convolution) and upsampling, gradually restoring the spatial resolution of the image and generating the final segmentation outcome. Consequently, when the encoder comprises eight layers (typically a combination of convolutional and pooling layers), the decoder correspondingly mirrors this with eight layers, preserving the overall symmetry of the network. This symmetry not only imparts a regular and intuitive structure that is easier to design and adjust but also facilitates the efficient transfer of feature information between the encoder and decoder, enhancing the model’s performance. The improved U-Net model features 16 layers. The input layer uses a 1 × 1 × 1,024 one-dimensional signal. Convolution is performed with a kernel size of 1 × 5 and a ReLU activation function in four-channel mode, followed by max pooling with a 1 × 4 pooling kernel. This produces an output of 1 × 32 × 256. The output is then downsampled again using a 1 × 5 convolution kernel and a ReLU activation function in 2-channel mode, resulting in a size of 1 × 64 × 64. Further convolution with a ReLU activation function in 2-channel mode is applied, followed by max pooling, producing an output of 1 × 128 × 16. This process is repeated with another convolution and ReLU activation function in 2-channel mode, followed by max pooling, resulting in 1 × 256 × 4. This completes the left side of the U-Net network model.

**Figure 2:**
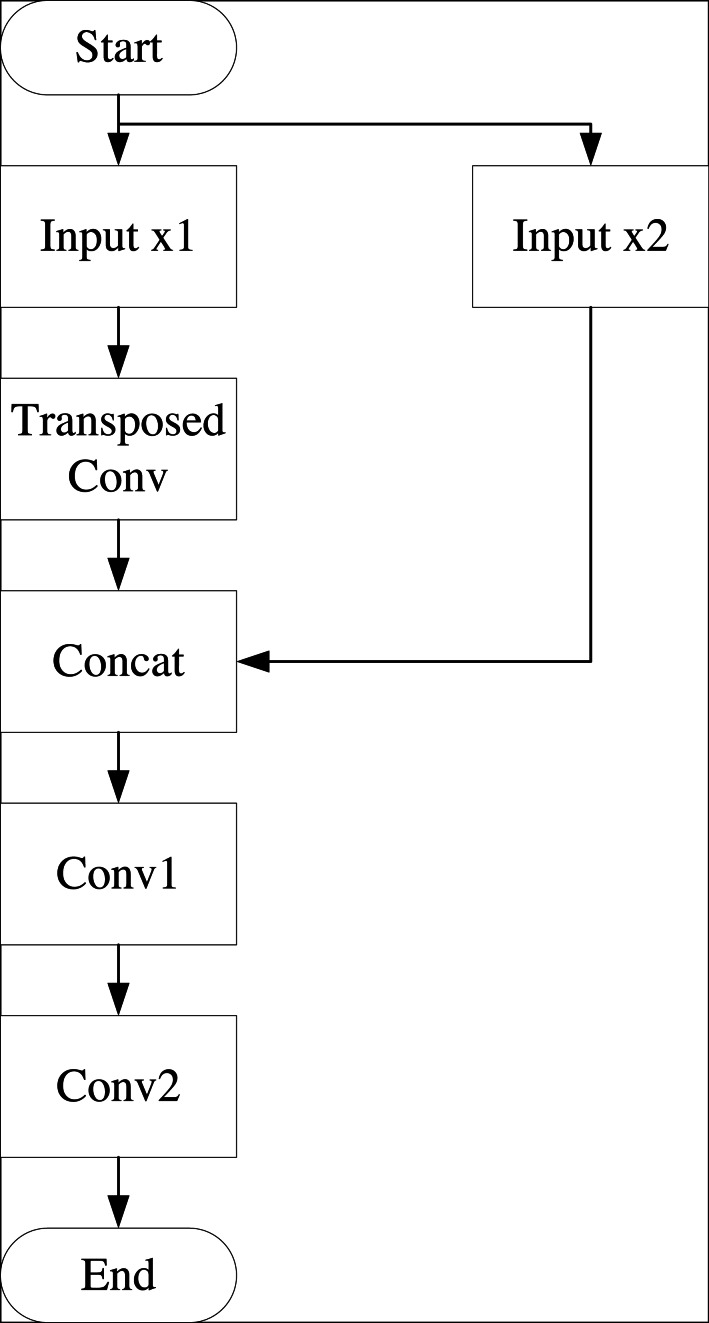
Deconvolution flowchart.

On the right side, the process begins with deconvolution, or up-sampling. The feature maps from deconvolution are merged with the symmetric feature maps from the left compression path. This merging involves convolution in 2-channel mode with a ReLU activation function, repeated twice. Deconvolution is then applied, followed by single-channel convolution, merging with feature maps from the left compression path to produce the final output with dimensions of 1 × 1 × 1,024. The flow of the deconvolution process is illustrated in Fig. 2.

### Fuzzy reinforcement *Q* learning

In fuzzy control, the process begins with a measuring device that captures state information, producing an exact deviation value. This value is then fuzzified to obtain fuzzy quantities. The fuzzy controller subsequently performs fuzzy reasoning based on predefined control rules to calculate the fuzzy control quantity, which is then used to adjust the exact quantity accordingly.

In the fuzzy reinforcement learning model, the fuzzy rule form is defined as follows:

(1) \begin{eqnarray*}{R}_{1}:~\mathrm{if}~{x}_{1}~\mathrm{is}~{F}_{1}^{1},\ldots ,{x}_{n}~\mathrm{is}~{F}_{1}^{n}, \text{then}{y}_{1}={c}_{0}^{1}+{c}_{1}^{1}{x}_{1}+\cdots +{c}_{n}^{1}{x}_{n}.\end{eqnarray*}

Where, ${F}_{1}^{i}$ is a fuzzy set, ${c}_{1}^{i}$ is a real number and *y*_*i*_ is the output of the system obtained by the approximation rule. For an input vector (*x*_1_, *x*_2_, …, *x*_*n*_)^*T*^, the output of the fuzzy system is calculated as follows.

(2) \begin{eqnarray*}y=\sum _{j=1}^{M}{y}_{j}\prod _{i=1}^{n}{\mu }_{{F}_{i}^{j}}({x}_{i}) \left/ \right. \sum _{j=1}^{M}\prod _{i=1}^{n}{\mu }_{{F}_{i}^{j}}({x}_{i}).\end{eqnarray*}

**Figure 3:**
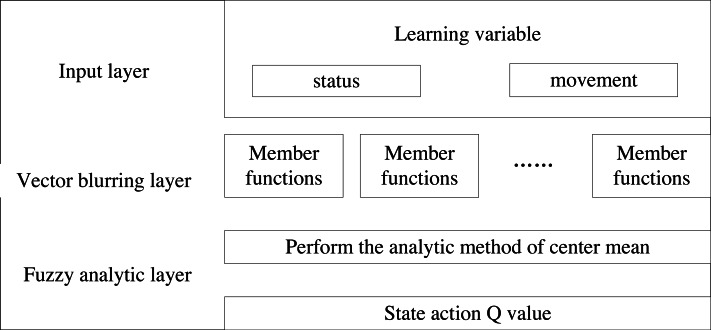
Fuzzy control model.

Next, based on the fuzzy control model, the fuzzy control result, which approximates the *Q*-value function, is calculated. The model is structured into three layers, as illustrated in Fig. 3.

The first layer is called the input layer and the main function of this layer is to receive learned notations of input variables:

(3) \begin{eqnarray*}x=({s}_{1},\ldots ,{s}_{u},a)^{T}.\end{eqnarray*}

The vector is constructed by combining the state vector and the action vector, with the dimension of the input vector maintained as u+1throughout the learning process. The state vector, representing the previous part of the state space, is denoted as:

(4) \begin{eqnarray*}s=({s}_{1},\ldots ,{s}_{u})^{T}.\end{eqnarray*}

The part of the vector that follows the vector is denoted as the action vector component.

The second layer is the layer where the fuzzification process of the vectors is carried out. Assume that the input component of the first layer is denoted as *x*_*i*_(*i* = 1, 2, …, *u* + 1), which includes a total of *J* members. To obfuscate each input component, we assign each member a differentiable elliptic Gaussian function (also called a membership function) as its member function. Suppose the j-th membership function of *x*_*i*_ is denoted as shown in Eq. (5).

(5) \begin{eqnarray*}M{F}_{ij}({x}_{i})=\exp \nolimits (-({x}_{i}-{u}_{ij})^{2}/{\sigma }_{ij}^{2}), (i=\{ 1,2,\ldots ,u+1\} ,j=\{ 1,2,\ldots ,J\} ).\end{eqnarray*}

Where *μ*_*ij*_ and *σ*_*ij*_ denote the center and width of the differentiable elliptic Gaussian function respectively. Next, we calculate the emission intensity of the fuzzy rule according to the membership function values of all members. Assuming K fuzzy rules, the emission intensity of Rule k (also known as activation or confidence) is calculated by multiplying all relevant input member functions as *φ*_*k*_(*x*):

(6) \begin{eqnarray*}{\varphi }_{k}(x)=\prod _{i=1}^{u+v}\exp \nolimits (-({x}_{i}-{u}_{ik})^{2}/{\sigma }_{ik}^{2}), (k=\{ 1,2,\ldots ,K\} ).\end{eqnarray*}

The third layer is the fuzzy parsing layer, which calculates the output state-action *Q*-values using the central mean parsing method:

(7) \begin{eqnarray*}{\delta }_{i}={\varphi }_{i} \left/ \right. \sum _{k=1}^{k}{\varphi }_{k}& \end{eqnarray*}

(8) \begin{eqnarray*}Q({s}^{T},{a}^{T})=\sum _{k=1}^{k}{w}_{k}{\delta }_{k}.& \end{eqnarray*}

Where *K* denotes the number of rules and *w*_*k*_ is the rule posterior.

### U-FRQL-EA

In a UAV cluster, when a UAV sensor node i acquires a packet P to be forwarded, it must select one of its neighboring nodes to forward the packet. This chosen node must be located within the target region R, which is identified by the center D. In this article, the node with the maximum value of the fuzzy computation result Q(*N*_*i*_) is selected as the forwarding node for the next hop.

Therefore, in UAV communication, after noise reduction has been applied to the communication channel, the set of neighboring nodes is evaluated. The neighbor node closest to the center of the region D within the entire sensor region is selected as the candidate forwarding node:

(9) \begin{eqnarray*}{F}_{i}=\text{node}\in {N}_{i}{|}d(i,D)-d(\text{node},D)> 0.\end{eqnarray*}

Where *d*(*i*, *D*) and *d*(node, *D*) denote the distance between this node and its neighboring nodes to the destination node in the target area, respectively. At this point every node is initialized with a Q value at the beginning of network operation. For the node node ∈ *F*_*i*_, the initialized Q value of node i is assumed to be Q(i), which is calculated as follows:

(10) \begin{eqnarray*}Q(i)=\alpha K/ \frac{1}{N} \sum _{\mathrm{node}\in {F}_{i}}d(\mathrm{node},D)+(1-\alpha )\mathrm{Max}(\mathrm{e}(i,\mathrm{node})).\end{eqnarray*}

Where *K* is a scaling factor which represents the percentage of energy overhead when the coordinates of the node performs packet forwarding. $ \frac{1}{N} {\sum }_{\mathrm{node}\in {F}_{i}}d(\mathrm{node},D)$ is the average distance to be traveled by node i to all its next hop neighboring nodes for forwarding packets. Max(e(*i*, node)) is the maximum value of energy overhead when node carries out data transmission with its neighboring nodes. The *Q*-value of each node is updated in real time based on the energy status of the node during the transmission of network data. The values of the neighboring nodes are also updated continuously.

When node *i* forwards a packet, there may be both successful and unsuccessful forwarding attempts. Upon receiving the packet, the node compares the *Q*-values of all its neighboring nodes, selecting the node with the highest *Q*-value as the forwarding node for the next hop. If the packet forwarding is successful, both node and its neighboring node receive a positive feedback factor to update their *Q*-values. Conversely, if the packet forwarding is unsuccessful, both node K and its neighboring node receive a negative feedback coefficient, resulting in a decrease in their *Q*-values.

In UAV cluster networks, packet forwarding failures can primarily occur for two reasons: (1) sending failures at the data node and (2) failures in selecting the appropriate next-hop neighbor node. If all nodes experience sending failures, it leads to network congestion. In such cases, if a node fails to forward the packet, it automatically reduces its fuzzy value to zero. Consequently, the neighboring nodes are updated based on the current fuzzy *Q*-values, and the node halts packet forwarding. This mechanism effectively mitigates network congestion and achieves dynamic balancing of the network’s energy resources.

## Experiments and Analysis

In this section, we analyze the performance of the improved U-Net model and U-FRQL-EA to verify the channel equalization capability of U-FRQL-EA in UAV communication.

### Experimental environment

To verify the effectiveness of the equalization algorithm proposed in this article, two sensor network simulation tools were utilized: the OMNeT++ simulation tool and the third-party model MobilityFramework2. These tools facilitate the random deployment of UAV communication nodes and offer the capability to set specific coordinate values for sensor placement. The sensors deployed in the network area are primarily responsible for data sensing and acquisition within designated target regions.

In the simulation, the following parameters were assumed: the maximum communication distance of each UAV node is set to 200 m, the communication radius of the base station node is 100 m, and the communication radius of each sensor node is 10 m. Additionally, the sensor’s transmission power and propagation sensitivity are calculated using the following formula:

(11) \begin{eqnarray*}{W}_{s} \left( dBm \right) +{W}_{sa} \left( dB \right) +{W}_{ra} \left( dB \right) -{W}_{rs} \left( dBm \right) -100~dB=20\ast \mathrm{log} \left( D \left( km \right) \right) .\end{eqnarray*}

Where *W*_*s*_, *W*_*sa*_, *W*_*ra*_ represent a set of coefficients describing the transmitting end, characterizing the transmitting power and the antenna gain of the transmitting end, respectively. The radius of the communication distance *W*_*rs*_ isset to D. Assuming that the absolute power W of the remote base station node and the sensing node in the network is both 20 mW (equivalent to 17 dBm), and the absolute power W of the sensing node in the network is set to 1 mW (equivalent to 8 dBm), with an antenna gain of 10 dB, the reception sensitivity of the base station node is calculated to be −55 dBm, while the reception sensitivity of the sensing node is −43 dBm.

In the context of network communication, the failure of message forwarding is often attributed to a multitude of factors, including network congestion, link failures, and inadequate processing capabilities of nodes. Within the scope of this research, the phenomenon of reduced node fuzziness values upon message forwarding failure can be addressed by adopting strategies such as the Random Early Detection (RED) algorithm and flow control mechanisms, in conjunction with existing classical methods, to tackle network congestion issues. This approach enables an effective management of congestion, mitigating its negative impact on message delivery and overall network performance.

### Evaluation index

In acoustic noise reduction for communication, the signal-to-noise ratio (SNR) and the mean square error (MSE) can effectively evaluate the noise reduction effect. Let *P*_signal_ and *A*_signal_ refer to the useful signal, *i.e.,* pure signal, for which the SNR is prepared. *P*_noise_ and *A*_noise_ refer to the pure noise. It can also be directly expressed as:

(12) \begin{eqnarray*}\mathrm{SNR}=10~\log \nolimits ~10({P}_{\mathrm{signal}}/{P}_{\mathrm{noise}})=20~\log \nolimits ~10({A}_{\mathrm{signal}}/{A}_{\mathrm{noise}}).\end{eqnarray*}

The SNR is the most intuitive measure of noise reduction effectiveness. A higher signal-to-noise ratio indicates that the signal contains less noise, thereby reflecting better noise reduction performance. The MSE quantifies the degree of discrepancy between the estimated quantity and the actual quantity. It is defined as the average of the squared differences between the filtered signal and the pure signal. The formula for calculating the mean square error is provided below:

(13) \begin{eqnarray*}\mathrm{MSE}= \left( \sum _{i=1}^{N}{|}{A}_{\mathrm{ signal}}-{A}_{\mathrm{denoised}}{{|}}^{2} \right) \left/ \right. N\end{eqnarray*}

where *A*_denoised_ represents the filtered signal.

### Signal processing capability analysis

In UAV communication systems, the channel noise characteristics are critical factors affecting the quality and reliability of data transmission. As UAVs typically operate in complex and diverse environments, such as urban landscapes, mountainous regions, and above oceans, their communication channels confront a multitude of noise challenges. These include multipath effect noise, where signal propagation through multiple paths caused by ground reflections, building scattering, and so on, results in rapid fluctuations due to phase superposition, thereby impacting the intensity and stability of the received signal. Additionally, atmospheric noise arises from variations in the ionosphere, troposphere, as well as weather conditions like rain, fog, and snow, all of which introduce additional noise, especially pronounced in high-frequency communications. Most significantly, electromagnetic interference noise, stemming from electromagnetic radiation from other wireless communication systems, radars, high-voltage power lines, and the like, can severely disrupt UAV communication links, degrading signal quality.

Therefore, in real-world scenarios, it is imperative to implement noise reduction measures for the signals. We must leverage advanced signal processing techniques and algorithms to mitigate the impact of these noise sources, ensuring robust and reliable communication for UAVs. The network is trained on collected data over 200 training rounds. The training dataset comprises 12,000 samples from four sensor collection points at 0 degrees, 90 degrees, 180 degrees, and 270 degrees. Following the training, the network model is validated with newly collected data. The effectiveness of the magnetic acoustic signal noise reduction is assessed through experimental evaluation metrics, specifically the signal-to-noise ratio and mean square error.

**Figure 4:**
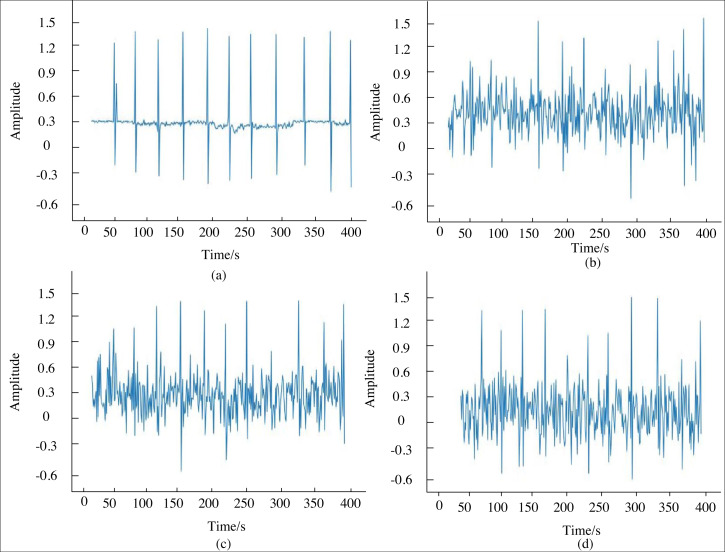
Contrast of de-noising waveform of magnetoacoustic signal.

In this section, experimental simulations are conducted using noisy magnetoacoustic signals, with an initial signal-to-noise ratio of −3.5725 dB and a mean square error of 0.3382 at 0 degrees, as obtained from laboratory measurements. Figure 4 illustrates the comparative denoising waveforms of the magnetoacoustic signals under the model. Specifically, (a) represents the waveform of the pure magnetoacoustic signal; (b) shows the denoised waveform at epoch 100; (c) depicts the denoised waveform at epoch 150; and (d) displays the denoised waveform at epoch 200. By comparing the acoustic signals across different training rounds, it is evident that the network model with 150 epochs achieves the most effective data denoising. At this epoch, the signal-to-noise ratio improves to 2.2738 dB, and the mean square error reduces to 0.0591. The improved U-Net model enhances the signal-to-noise ratio by 5.8312 dB and decreases the mean square error by 0.2712, demonstrating the model’s effectiveness in processing magnetoacoustic signals.

### Energy consumption performance analysis

**Figure 5:**
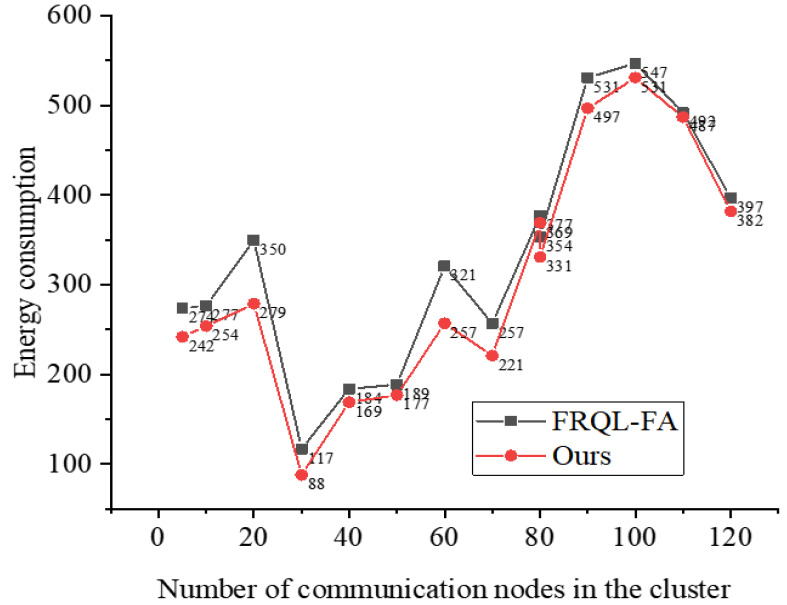
Optimize energy consumption data of sensor nodes before and after communication.

After confirming the improvement in noise reduction performance through signal processing, we evaluate energy consumption under the same noise conditions to ensure consistency in the test environment, thereby allowing for direct and accurate comparisons of algorithm performance. This approach helps eliminate potential interference from varying noise conditions on the assessment results, making the outcomes more reliable and reproducible. Therefore, the monitoring area is defined as a two-dimensional plane measuring 3,000 m × 3,000 m, with a communication radius of 50 m. The setup includes 120 sensing nodes, each with a communication radius of 10 m. Data is deployed using an as-you-go scattering approach. When a sensing node’s memory space approaches saturation, it transmits the data to the base station. The node employs a data fusion ratio of 1:100. The results of the simulation are presented in Fig. 5.

Figure 5 provides a comprehensive comparison of the energy consumption characteristics between two key algorithms: U-FRQL-FA and its predecessor, FRQL-FA, which does not incorporate the improved U-Net model. The performance of tFRQL-FA model has been rigorously verified through a vast array of research studies and practical applications. In order to fortify the persuasiveness and credibility of our conclusions, we have deliberately chosen this model as our benchmark for comparison. This comparative analysis highlights the innovative advantages of the U-FRQL-FA algorithm, particularly its integration of the deeply optimized U-Net model. As a fundamental component of signal processing, the U-Net model effectively filters out noise interference in acoustic signals and significantly enhances the system’s adaptive sensing capability in complex and fluctuating communication environments. Consequently, U-FRQL-FA demonstrates exceptional precision and flexibility in dynamically adjusting communication strategies and optimizing resource allocation.

**Figure 6:**
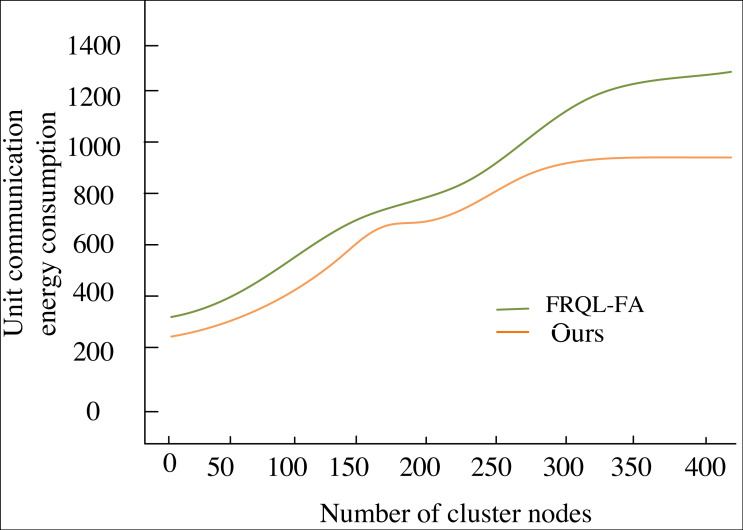
Communication energy consumption curve.

Upon examining the data trends in Fig. 6, it is evident that the energy-saving advantages of the U-FRQL-FA algorithm become increasingly pronounced as the number of sensor network nodes grows. The algorithm achieves a refined optimization of the equalization process through the innovative integration of deep learning and reinforcement learning techniques. This results in a substantial reduction in energy consumption per communication unit, with the savings in energy loss compared to the unoptimized FRQL-FA algorithm widening as data volume increases. This trend is visually represented in the graph as a smooth, continuously downward curve, highlighting the superior performance of U-FRQL-FA in energy efficiency management. Notably, while the early advantage of U-FRQL-FA in energy savings may appear modest due to the system’s initialization and debugging phase, this transient effect does not diminish the algorithm’s long-term efficacy. As the UAV trunked communication network expands and the number of nodes increases, coupled with the stabilization of the network layout, the optimized algorithm demonstrates its robust adaptive capability and efficient resource management strategy. The pronounced downward trend in the latter part of the curve serves not only as strong evidence of the algorithm’s performance but also as a compelling validation of the research methodology presented in this article.

### Comparative analysis

**Figure 7:**
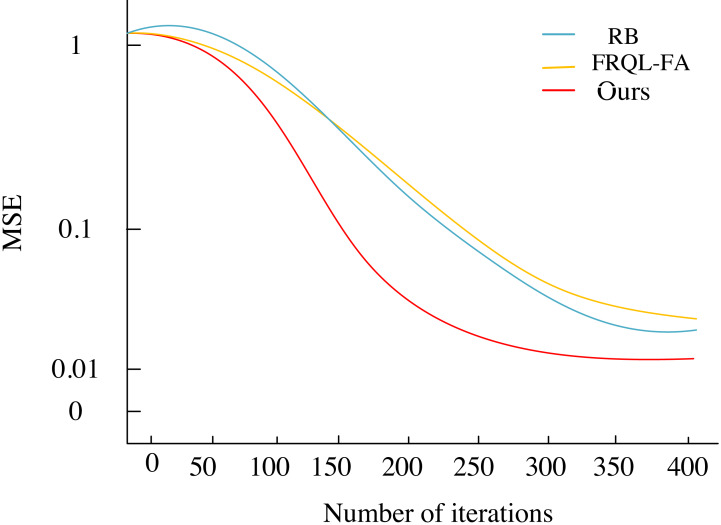
Convergence contrast.

This section analyzes the equalization performance of the U-FRQL-FA algorithm by comparing it with the RBF algorithm from the literature  (Chew, He & Zhang, 2024) andthe CNN-FA algorithm from the literature (Zhao et al., 2024), using the environment settings described in ‘Experimental Environment’. Faster convergence speed is indicative of a shorter time required for convergence and a lower number of bit error rates (BERs) at the beginning of communication, reflecting better communication quality. Conversely, slower convergence results in poorer communication quality. Figure 7 illustrates the convergence plots of the mean square error for each equalization algorithm after iteration. As shown in Fig. 7, the proposed U-FRQL-FA algorithm achieves the desired training target with approximately 250 iterations, demonstrating superior convergence speed. In contrast, both the RBF algorithm and the CNN-FA algorithm exhibit significantly slower convergence, with noticeable convergence tendencies only occurring after around 350 iterations.

**Figure 8:**
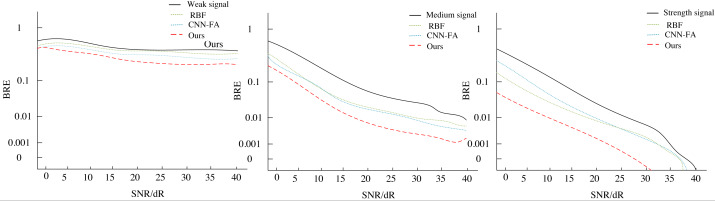
The BER of different models.

Figure 8 illustrates the variation in system bit error rate (BER) with SNR for different communication signals. As depicted, the incorporation of the blind equalization algorithm proposed in this paper, which integrates U-Net and fuzzy reinforcement learning for noise reduction and adaptive sensing, leads to notable improvements in the BER of the communication system across weak, medium, and strong signals. Firstly, under low SNR conditions, our proposed algorithm achieves the same BER level at a significantly lower SNR threshold (approximately 3 to 4 dB improvement) compared to the RBF and CNN-FA algorithms. This underscores the algorithm’s superior ability to effectively suppress noise in harsh communication environments, enabling it to reach below the forward error correction (FEC) threshold for BER earlier, hence demonstrating faster convergence. In environments characterized by medium to strong signal strengths, even when signal quality is inherently good, our algorithm continues to exhibit remarkable performance efficiency. Notably, at an SNR of 10 dB, the BER falls below the FEC tolerance limit, highlighting the system’s high adaptability and stability across a wide range of signal intensities. Consequently, as the SNR improves, the algorithm converges to a lower BER state even more rapidly.

## Conclusion

This paper proposes the U-FRQL-EA equalization algorithm, which leverages adaptive perception and reinforcement learning to meet the demands of UAV communication environments. The U-FRQL-EA algorithm incorporates U-Net for preprocessing, which significantly enhances the signal-to-noise ratio by reducing noise in the received signal. This approach effectively addresses the issue of signal degradation due to noise interference during transmission in UAV communication systems. Particularly in low SNR conditions, experimental results demonstrate that this method substantially mitigates the impact of noise on signal quality, providing high-quality input data for subsequent adaptive equalization. Given the complexity and variability of UAV communication channels, traditional equalization algorithms struggle to adapt to real-time channel changes. To address this, fuzzy reinforced *Q*-learning is introduced to achieve adaptive perception of channel characteristics, dynamically adjusting the equalizer tap coefficients in the blind equalization algorithm. This enables a rapid response to channel changes and facilitates adaptive equalization. The U-FRQL-EA equalization algorithm effectively reduces system BER and improves communication quality across varying signal strengths, as confirmed by simulation experiments. Future work will focus on further optimizing algorithm parameters and model structures to handle more complex UAV communication scenarios and meet higher performance requirements.

Incorporating the experimental training process that examines the system’s BER variation against SNR across balanced performance and diverse communication signals, this paper harnesses the parallel computing capabilities of the GPU platform to accelerate the training process, achieving approximately an 80% reduction in U-Net’s training time compared to traditional CPU-based training. Additionally, the employment of Batch Normalization and Rectified Linear Unit (ReLU) activation functions expedited model convergence while mitigating the issue of vanishing gradients, leading to a more rapid decline in the loss function during training.

To approximate the *Q*-value function, an efficient fuzzy logic system was leveraged. In comparison to traditional lookup table methods, this approach ensures comparable approximation accuracy (with errors contained within ±0.05 range) while significantly reducing storage requirements and computation time. The utilization of the fuzzy logic system translated to a roughly 60% decrease in computational overhead per decision-making instance.

Ultimately, as depicted in Fig. 8, the system, through U-Net’s noise reduction processing and the adaptive decision-making capabilities of fuzzy *Q*-learning, successfully reduced the BER by approximately three orders of magnitude, dropping from initially above 10 $\hat {}$-2 to below 10 $\hat {}$-5. Notably, at an SNR of 11dB, the BER fell below the threshold of Forward Error Correction (FEC) coding, demonstrating remarkable noise resilience. Even in medium to strong signal environments, the system maintained high performance, with BER continuously decreasing as SNR increased. Specifically, when SNR reached 10dB, the BER surpassed the FEC limit, further validating the algorithm’s efficiency and stability.

##  Supplemental Information

10.7717/peerj-cs.2557/supp-1 Code S1 Code

## Additional Information and Declarations

### Data Availability

The following information was supplied regarding data availability: The data is available at Zenodo: Skriver M. 2022. UAV exposed to Ultra Bandwidth Noise Recorded using HEIST (1.0) [Data set]. Zenodo. https://doi.org/10.5281/zenodo.6573631.
